# IMPLEMENTATION STUDY OF THE USE OF A BLOOD BIOMARKER TEST IN ALZHEIMER’S DISEASE: CLINICIAN SURVEY FROM QUIP II STUDY

**DOI:** 10.1093/geroni/igae098.4301

**Published:** 2024-12-31

**Authors:** Mark Monane, Robert Carlile, Kim Johnson, Darren Gitelman, Lawren VandeVrede, Leslie Jacobs, Joel Braunstein

**Affiliations:** C2. N Diagnostics, St. Louis, Missouri, United States; Palmetto Primary Care Physicians, Summerville, South Carolina, United States; Duke University, Durham, North Carolina, United States; Advocate Lutheran General Hospital, Park Ridge, Illinois, United States; UCSF, San Francisco, California, United States; C2. N Diagnostics, St. Louis, Missouri, United States; C2. N Diagnostics, St. Louis, Missouri, United States

## Abstract

Recent studies have shown that commercially available, high-performing blood biomarkers (BBMs) for Alzheimer’s disease (AD) pathology represent accurate, accessible, acceptable, and equitable tools to aid health care providers (HCPs) in the evaluation of their patients with signs or symptoms of mild cognitive impairment (MCI) or dementia. However, implementation of AD BBM tests into the clinical care pathway has not been extensively evaluated. The objective of this study was to assess the incorporation of the multi-analyte blood biomarker PrecivityAD2™ test (C2N Diagnostics, LLC, St. Louis, MO) into the clinical workflow of specialty clinics. A total of 203 patients (average age 74 years) evaluated by 12 HCPs (neurologists, geriatricians, geriatric psychiatrists, and others) representing 8 outpatient sites were included as part of the real-world QUIP II Study (NCT06025877). A five-item, end-of-study survey was developed and conducted using The Technology Acceptance Model (David, 1989) and included two constructs: perceived usefulness and perceived ease of use of this BBM test. HCP-reported acceptance scores averaged 9.6 (median 10, range 7-10): contribution to clinical decision-making and ease of understanding test results received the highest ratings. The net promoter score was 75, above the benchmark of 50 associated with excellent customer satisfaction (Qualtrics, 2024). Given recent guidelines and workshop recommendations highlighting the use of AD BBM tests in clinical care, we believe these survey data provide evidence of robust usefulness and acceptance of this BBM test, supporting its incorporation into the clinical care pathway to help HCPs rule in and rule out AD in cognitively symptomatic patients.

